# Mortality rates among individuals diagnosed with hepatitis C virus (HCV); an observational cohort study, England, 2008 to 2016

**DOI:** 10.2807/1560-7917.ES.2019.24.30.1800695

**Published:** 2019-07-25

**Authors:** Georgina Ireland, Sema Mandal, Matthew Hickman, Mary Ramsay, Ross Harris, Ruth Simmons

**Affiliations:** 1 National Infection Service, Public Health England, London, United Kingdom; 2 The National Institute for Health Research Health Protection Research Unit (NIHR HPRU) in Blood Borne and Sexually Transmitted Infections at University College London, United Kingdom; 3 The National Institute for Health Research Health Protection Research Unit (NIHR HPRU) in Evaluation of Interventions at University of Bristol, Bristol, United Kingdom; 4 Population Health Sciences, Bristol Medical School, Bristol, United Kingdom

**Keywords:** hepatitis C, mortality, direct acting antivirals, England, surveillance, epidemiology

## Abstract

**Background:**

Monitoring trends in mortality for individuals diagnosed with hepatitis C virus (HCV) infection are important as we expand treatment and move towards World Health Organization elimination targets.

**Aim:**

To estimate mortality rates for individuals aged ≥ 15 years diagnosed with HCV infection in England 2008–16.

**Methods:**

An observational cohort study whereby death certificate information was linked to the Sentinel Surveillance of Blood Borne Virus Testing in England. Age-sex standardised mortality rates (ASMR) for individuals diagnosed with HCV infection (2008–16) were calculated and compared to the general population.

**Results:**

Of 43,895 individuals with HCV infection, 2,656 (6.3%) died. All-cause ASMRs were 2,834.2 per 100,000 person years (PY), 2.3 times higher than in the general population. In individuals aged 30–69 years, all-cause mortality rates were 1,768.9 per 100,000 PY among individuals with HCV, 4.7 times higher than in the general population. ASMRs had not decreased between 2010 (2,992) and 2016 (2,340; p=0.10), with no change from 2014 (p = 0.058). ASMRs were 441.0 times higher for hepatitis, 34.4 times higher for liver cancer, 8.1 times higher for end stage liver disease and 6.4 times higher for external causes than in the general population.

**Conclusions:**

Mortality was higher in individuals with diagnosed HCV infection compared to the general population, highlighting health inequalities. There is a need to improve HCV diagnosis, engagement in care and treatment rates. The high mortality from external causes highlights the importance of integrated health and social care strategies and addressing the needs of this vulnerable population.

## Introduction

In England, an estimated 0.4% (160,000) of the population have chronic hepatitis C virus (HCV) infection, with the main group at risk of HCV infection being persons who inject drugs (PWID) [1]. Individuals with chronic HCV infection are at increased risk of liver cirrhosis, end-stage liver disease (ESLD) and hepatocellular carcinoma (HCC), the burden of which have been increasing over the past 10 years [2].

Historically, HCV treatment rates in England have been low, however, the treatment landscape for individuals with chronic HCV infection has dramatically changed following the introduction of direct-acting antivirals (DAAs) in 2014, which have a shorter treatment duration, are better tolerated and are more effective than previous interferon-based regimens [3]. Successful curative treatment, defined as a sustained virological response, improves the outcomes in individuals with HCV infection including reduced mortality rates [4-6]. With these changes to treatment it is important to understand the burden of disease and associated mortality in the pre-DAA era. This will enable us to effectively monitor their impact on the cascade of care (including mortality) taking into account the increased efforts to diagnose, engage in care and treat individuals with HCV infection. This is particularly relevant as countries across Europe, including the United Kingdom (UK), are working towards the World Health Organization (WHO) global strategy for elimination of viral hepatitis as a significant public health threat by 2030 and reduction in HCV-associated deaths is one of the metrics for monitoring progress [7].

Using information on testing from the Sentinel Surveillance of Blood Borne Virus Testing (SSBBV) and Office for National Statistics (ONS) Death Registry in England, we aim to estimate mortality rates for individuals (≥ 15 years) first reported to SSBBV with diagnosed HCV infection between 2008 and 2016 and describe the characteristics of changes that have occurred following the introduction of DAAs in 2014.

## Methods

### Study design

This is an observational cohort study looking at HCV-related mortality rates in individuals aged 15 years or older, diagnosed with HCV infection between 2008 and 2016 in England.

### Data sources

#### Sentinel Surveillance of Blood Borne Virus Testing (SSBBV)

The sentinel Surveillance of Blood Borne Virus Testing (SSBBV), held at Public Health England (PHE), collects hepatitis A-E, HIV and Human T-lymphotropic virus (HTLV) testing information from 23 participating laboratories in England. Information on tests is collected regardless of result and includes demographics (age, sex and ethnicity) and the service requesting the test [8,9]. Multiple tests relating to the same person are linked using a combination of soundex (phonetic algorithm for indexing names), date of birth, National Health Service (NHS) number and hospital number. Participating laboratories are broadly representative of England as a whole and cover ca 40% of the registered general practitioner (GP) population in England. When possible, the date of first HCV infection diagnosis in SSBBV is updated by the routine laboratory reports of HCV and Hospital Episode Statistics (HES). Diagnosis dates prior to those reported in SSBBV are identified in HES where an earlier inpatient stay is recorded with viral hepatitis ICD-10 diagnosis codes (B17.1 and B18.2). The routine laboratory reports of HCV and HES were linked to SSBBV using NHS number, sex and date of birth. Following linkage and date of diagnosis improvement, individuals with a diagnosis date for HCV infection before 2008 were excluded.

#### Office for National Statistics (ONS) cause of death registry

The registry of deaths at ONS, includes all reported deaths in England since 1937 [10]. Cause of death is coded according to the tenth revision of the International Statistical Classification of Diseases and Related Health Problems (ICD10 [11]) as underlying cause or as one of nine contributory causes.

### Data collection and linkage

In 2018, all individuals aged 15 years or older diagnosed with HCV infection and first reported to SSBBV between 2008 and 2016 were extracted from SSBBV. Individuals were excluded if they did not have date of birth or sex recorded. NHS laboratories in England submit positive HCV test results (predominantly anti-HCV, some HCV RNA) to the routine laboratory reporting of HCV at PHE. The system does not distinguish between anti-HCV and HCV RNA positivity and so laboratory ‘confirmed’ cases are a mix of current and ever infected individuals. Data have been collected since 1990, and since 2010, HCV infection has been a notifiable causative agent and all diagnostic laboratories are legally required to report new diagnoses to PHE. Data completion varies over time.

In April 2018, ONS provided all reported deaths between 2008 and 2016 in individuals aged 15 years or older living in England. Underlying cause of death ICD10 codes were grouped according to Table 1. In addition to the groupings we looked at: (i) all-liver disease mortality (underlying), by grouping liver cancer, ESLD, viral hepatitis, alcoholic and non-alcoholic liver disease diagnostic codes, and (ii) all-liver disease mortality (underlying and contributory), where liver cancer, ESLD, hepatitis, alcoholic and non-alcoholic liver disease were recorded as either the underlying or contributory (i.e. as any of the additional nine causes) cause of death.

**Table 1:** Primary cause of death groupings, with ICD10 codes

Cause of death grouping	IDC10 Codes
Viral hepatitis	B15–19
End stage liver disease	I850, I983, K704, K720, K721, K729, K767, R18
Liver cancer	C22
Alcoholic liver disease	K70, excluding K704
Non-alcoholic liver disease	K71-K77, excluding K720, K721, K729, K767
External causes	S00–99, T00–98, V01–99, W00-W99, X00-X99, Y00-Y98
Other	All other ICD-10 codes

SSBBV and the registry of deaths were linked using a deterministic approach i.e. the data were first linked using NHS number in conjunction with date of birth (then dropping either the day, month or year), the data were then linked with soundex (a phonetic algorithm for indexing names) and initial before finally being linked with soundex and year of birth. In instances where the NHS number was not available, we matched on soundex, initial and date of birth. A hierarchical approach was used for each year where matches by NHS number superseded a match without an NHS number. Additional checks were conducted on records where a person had been tested after their date of death, where a person has been linked to a death recorded in a previous year and where multiple records matched.

### Statistical analysis

Overall and disease-specific mortality rates per 100,000 person years (PY), standardised to the 2013 European standard population by age and sex, were calculated for all individuals (≥ 15 years) and within a subset of individuals aged 30–69 years (premature mortality). ASMRs for males and females are age-standardised only. Confidence intervals (CI) were calculated using the Poisson distribution [12]. Individual follow-up began 6 months after HCV diagnosis and ended at death (between 2008 and 2016) or 31 December 2016.

Individuals who died within 6 months of their HCV diagnosis were excluded from the analysis, as there was potential bias towards higher rates of diagnosis in individuals with major morbidity. Annual age-sex adjusted mortality rates (ASMRs) were calculated for the period 2008 to 2016, allowing trends to be explored for all-cause, underlying and contributory all-liver disease mortality. The small number of people with HCV (who died) in 2008 and 2009 (due to very short follow-up times from diagnosis) led to unstable rates for the first 2 years of analysis and therefore ASMRs for these years were excluded when presenting trends. Joinpoint Regression Programme Version 4.2.1.0 (National Cancer Institute, Bethesda, United States) was used to analyse for changes in ASMRs at 2014, the year when DAAs were introduced in England. Joinpoint can identify statistically significant changes in trend data over time and whether there was a point at which a statistically significant change in the trend occurred [13].

For comparison, ASMRs for the general population were estimated using all death reports reported to ONS for 2012 divided by the mid-2012 population estimates, the mid-point of our analysis period. Mortality rate ratios were calculated by dividing the ASMR among individuals diagnosed with HCV infection between 2008 and 2016 by the ASMR among the English population in 2012 and 95% CI were calculated by dividing the ASMR 95% CI for individuals diagnosed with HCV by the ASMR for England.

### Ethics

The data were collected for the surveillance of HCV infection, including linkage to death registrations held by the Office for National Statistics and is covered by Health Service (Control of Patient Information) Regulations 2002 (regulation 3).

## Results

During the study period (2008–16), of 43,895 individuals (≥ 15 years) diagnosed with an HCV infection reported to SSBBV, 2,656 (6.3%) had died. Total follow-up time was 183,821 PY and median follow-up time was 4.1 years (interquartile range (IQR): 2.1–6.3 years). The majority of individuals with HCV infection were male (67.6%) and the median age at diagnosis was 39 years (IQR: 32–48 years) (Table 2). Of 2,656 individuals who died within the follow-up period, the median time from diagnosis to death was 3 years (IQR: 1.6–4.7 years).

**Table 2:** Characteristics of individuals diagnosed with HCV infection in SSBBV, England, 2008–2016 (n = 43,895)

Characteristics	Total		Deaths	
	n	%	n	%
	43,895	100	2,656	6.1
**Sex**				
Male	29,671	67.6	1,914	6.5
Female	14,224	32.4	742	5.2
** Age**				
15–29	8,007	18.2	143	1.8
30–39	14,415	32.8	494	3.4
40–49	12,225	27.9	733	6.0
50–69	8,228	18.7	948	11.5
70 +	1,020	2.3	338	33.1
**Ethnicity**				
White	28,599	65.2	2,311	8.1
Asian	3,984	9.1	200	5.0
Black	828	1.9	77	9.3
Other	1,249	2.8	44	3.5
Unknown	9,235	21.0	24	0.3
**Diagnosis year**				
2008–10	16,122	36.7	1,554	9.6
2011–13	15,545	35.4	874	5.6
2014–16	12,228	27.9	228	1.9

HCV: hepatitis C virus; SSBBV: Sentinel Surveillance of Blood Borne Virus Testing.

All-cause ASMR per 100,000 among individuals with HCV infection were 2,834.2 per 100,000 PY (Table 3), 2.3 times higher than that for the general population (1,218.5 per 100,000 PY), and were higher in males than females (3,170.7 vs 2,497.7 per 100,000 PY). A non-significant decrease in ASMRs was observed from 2,523.4 per 100,000 PY (95% CI: 1,801.6–3,245.2) in 2010 to 2,438.2 per 100,000 PY (95% CI: 2,106.8–2,769.6) in 2016 (p = 0.30) (Figure). When examining the possibility of a change in trend in 2014, the year DAAs became available on the NHS, there was no significant difference between the two slopes of best fit (p = 0.34).

**Table 3:** All-cause, sex-specific and disease-specific ASMR, per 100,000 person years, for individuals diagnosed with HCV infection, England, 2008–2016

Characteristics		England		Diagnosed with HCV infection						Mortality rate ratio	
		ASMR	95% CI	PY	Deaths		Crude mortality rate	ASMR	95% CI	Mortality ratio	95% CI
**Age ≥ 15 years**											
** Total**		**1,218.5**	**1,214.9–1,222.1**	**183,821**	**2,656**		**1,444.9**	**2,834.2**	**2,664.2–3,004.2**	**2.3**	**2.2–2.5**
** Sex**											
Male		1,391.1	1,385.2–1,397.0	123,022	1,914		1,555.8	3,170.7	2,927.2–3,414.1	2.3	2.1–2.5
Female		1,045.9	1,041.7–1,050.1	60,799	742		1,220.4	2,497.7	2,260.4–2,735.0	2.4	2.2–2.6
**Cause of death**											
Liver Cancer		10	9.6–10.3	183,821	233		126.8	344.4	283.8–405.1	34.5	28.5–40.6
ESLD		3.4	3.3–3.6	183,821	63		34.3	27.5	18.3–36.8	8.0	5.3–10.7
Viral hepatitis		0.5	0.5–0.6	183,821	207		112.6	220.5	175.8–265.3	421.9	336.3–507.6
Non-alcoholic liver disease		6.5	6.2–6.7	183,821	100		54.4	76.6	54.0–99.2	11.8	8.3–15.3
Alcoholic liver disease		7.4	7.1–7.7	183,821	238		129.5	122.0	100.4–143.6	16.5	13.6–19.4
All-liver disease (underlying)		27.8	27.3–28.3	183,821	841		457.5	791.1	709.0–873.2	28.4	25.5–31.4
All-liver disease (underlying and contributory)^a^		28.5	27.9–29.0	183,821	1213		659.9	1145.1	1,044.9–1,245.2	40.2	36.7–43.7
External causes		35.8	35.2–36.4	183,821	536		291.6	221.2	193.4–248.9	6.2	5.4–6.9
Other		1,154.8	1,151.3–1,158.4	183,821	1279		695.8	1821.9	1,675.7–1,968.1	1.6	1.5–1.7
**Age 30–69 years**											
**Total**	**377.9**		**375.5–380.2**	**161,622**		**2165**	**1,339.5**	**1,768.9**	**1,672.3–1,865.5**	**4.7**	**4.4–4.9**
** Sex**											
Male	454.8		451.1–458.5	111,618		1,639	1,468.4	2,016.5	1,978.4–2,234.7	4.4	4.3–4.9
Female	300.9		298.0–303.9	50,005		517	1,033.9	1,431.3	1,286.7–1,575.8	4.8	4.3–5.2
**Cause of death**											
Liver Cancer	5.2		5.0–5.5	161,622		175	108.3	206.8	169.8–243.7	39.5	32.4–46.5
ESLD	4.2		4.0–4.5	161,622		60	37.1	37.7	26.4–49.1	8.9	6.2–11.6
Viral hepatitis	0.6		0.5–0.7	161,622		173	107.0	165.2	134.0–196.4	263.6	213.8–313.4
Non-alcoholic liver disease	5.1		4.9–5.4	161,622		91	56.3	76.4	56.5–96.3	14.8	11.0–18.7
Alcoholic liver disease	10.1		9.7–10.5	161,622		232	143.5	169.4	142.8–196.0	16.8	14.1–19.4
All liver-disease (underlying)	25.4		24.8–26.0	161,622		731	452.3	655.5	595.8–715.3	25.8	23.5–28.2
All liver-disease (underlying and contributory)^a^	36.4		35.7–37.1	161,622		1,049	649.0	917.8	847.1–988.4	25.2	23.3–27.1
External causes	24.5		23.9–25.1	161,622		499	308.7	274.2	244.8–303.6	11.2	10.0–12.4
Other	328.1		325.9–330.2	161,622		926	572.9	839.1	769.2–909.1	2.6	2.3–2.8

ASMR: age and sex-standardised mortality rates; CI: confidence interval; ESLD: end stage liver disease; HCV: hepatitis C virus; PY: person years.

^a^ Individuals with liver cancer, ESLD, viral hepatitis, alcoholic liver disease and non-alcoholic liver disease recorded as an underlying cause, or any of the additional nine causes recorded.

**Figure:**
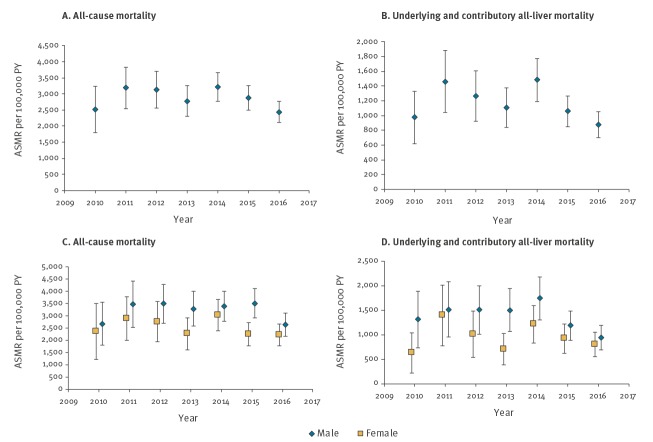
ASMR by all-cause mortality and underlying and contributory all-liver mortality for individuals aged 15 years and over, England, 2010–2016

ASMR: Age and sex standardised mortality rates; PY: person years.

ASMRs for individuals with a HCV infection diagnosis were higher than those in the general population for all grouped underlying causes of death. Of note, mortality rate ratios indicate that compared with the general population, individuals with HCV infection were 421.9 times more likely to die from viral hepatitis, 34.5 times more likely from liver cancer, 8.0 times more likely from ESLD, 16.5 times more likely from alcoholic liver disease, 11.8 times more likely from non-alcoholic liver disease and 6.2 times more likely to die from external causes than the general population. When deaths from liver disease were grouped, ASMRs for underlying all-liver disease and underlying and contributory all-liver disease were 28.4 and 40.2 times higher, respectively, than for the general population. A non-significant decrease in ASMRs for underlying and contributory all-liver disease was observed, from 975.0 (95% CI: 619.1–1,331.0) in 2010 to 875.4 (95% CI: 395.9–1,054.9) in 2016 (p = 0.3).

### Premature mortality

In individuals aged 30–69 years, all-cause ASMR was 1,768.9 per 100,000 PY among individuals with a HCV infection diagnosis, 4.7 times higher than the general population (England ASMR: 377.9 per 100,000 PY) (Table 3).

ASMRs per 100,000 PY in individuals aged 30–69 years were higher in men than women (2,016.5 vs 1,431.3 per 100,000 PY) and when compared with the general population. In this age group, ASMRs for all grouped causes of death were 263.6 times higher for viral hepatitis, 39.5 times higher for liver cancer, 16.8 times higher for alcoholic liver disease, 14.8 times higher for non-alcoholic liver disease, 8.9 times higher for ESLD and 11.2 times higher for external causes. ASMRs from underlying all-liver disease and underlying and contributory all-liver disease were 655.5 and 917.8 per 100,000 PY, respectively; 25.8 and 25.2 times higher per 100,000 PY than in the general population.

## Discussion

ASMRs for individuals diagnosed with HCV in England between 2008 and 2016 were more than two times higher than that found in the general population with higher rates in men than women. Mortality rates were between 8.0 and 421.9 times higher in individuals with HCV infection diagnosis for liver causes (ESLD, liver cancer, viral hepatitis, alcoholic and non-alcoholic liver disease) than for the general population and rates were 28.4 and 40.2 times higher, respectively, when underlying and contributory causes of death were included. Mortality rates for external causes were 6.2 times higher than in the general population and more pronounced in individuals aged 30–69 years. This may indicate a higher risk of premature mortality not only from liver related causes but also from overdose, intentional self-harm and accidents.

### Limitations

A main limitation is that we were only able to analyse individuals diagnosed with HCV infection between 2008 and 2016, with the maximum follow-up time being 8.5 years, but HCV-associated disease, particularly ESLD, can take 20-30 years to develop following acquisition of infection [14]. Whilst there is limited literature on time from acquisition of HCV infection to HCV diagnosis, it is thought to be at least 10 years in PWID [15]. Our mortality rates, therefore, may not be representative of all individuals with HCV infection in England and maybe skewed towards those with more advanced HCV-related disease presenting with complications and these individuals may be more likely to be tested for HCV as a result. We do not have disease stage data to adjust for this potential bias, but linkage of routine laboratory reports of HCV infection to liver transplant registries showed a similar short duration to transplant registration. This bias may be reduced in our study through a variety of different means. First, it was not possible to include HCV RNA testing information with 25% of anti-HCV positive individuals having no HCV RNA test recorded in SSBBV; we would expect mortality rates in individuals with treated or cleared HCV infection to be lower than individuals currently infected. Second, SSBBV is derived from a network of laboratories that cover 40% of the England GP-registered population. It is possible that we may not have identified a person’s initial HCV test if they had tested outside the sentinel sites or before SSBBV. However, our cohort was also linked to routine laboratory reports of new diagnoses (established in 1996) and HES inpatient data (established in 2004) so any earlier diagnosis i.e. before SSBBV started, should have been identified. In addition, while coverage varies by region, the network is broadly representative for England and our analysis allowed inclusion of more people e.g. those testing positive but not necessarily referred or engaged in care, improving representativeness of our data. Third, there can be delays in death registrations, due to coroners’ inquests into cause of death being required. Around 5% of all deaths are not registered in the year they occurred, rising to 50% for deaths relating to drug poisonings. Our mortality rate estimates for external causes in more recent years might be an underestimate, therefore, but it is unlikely to impact liver disease [16].

### Other evidence and implications

Our all-cause ASMRs were similar to those found in a study by Aspinall et al. based in Scotland [17]. There were some methodological differences (they calculated ASMRs for all individuals aged 1 years and over) and our mortality rates for liver cancer and alcohol associated liver disease were higher; this may be due to our larger sample size and only including individuals aged 15 years and over. We also found differences in ASMRs between males and females. This is likely to be associated with differences in co-morbidities e.g. smoking, alcohol and drug use [18,19], higher rates of spontaneous clearance of HCV and slower disease progression in females [20,21], which we were unable to adjust for. Alcohol misuse in HCV infected individuals can accelerate liver disease progression, which may explain the 15 times higher mortality rates from alcoholic liver disease [14,22].

Analysis with Joinpoint did not show a statistically significant decrease in all-cause and underlying and contributory all-liver disease mortality following the introduction of HCV treatment with DAAs in 2014; however the data does suggest that mortality rates have started to fall. Unlinked analysis of mortality records in England found a 3% reduction in the number of deaths from HCV-related ESLD and liver cancer in 2016 and a further 11% fall in 2017 [23], which have been largely attributed to improved access to new treatments. However, under-reporting of HCV infection remains an issue in mortality records [24-26]. The impact of DAAs and cascade of care improvements on mortality rates, will likely be more pronounced over time, as treatment restrictions are relaxed to include patients with little or no liver disease – thus preventing progression to severe liver disease with lower SVRs and higher mortality risk. A small rise in mortality rates in 2014 was notable, which although not significant, might have occurred due to patients deferring treatment with interferon-based regimes until they became eligible for treatment with DAAs.

When compared with England’s general population we found all-cause mortality rates were 2.3 times higher for all adults and 4.6 times higher for individuals 30–69 years old with a HCV infection diagnosis. These high rates of premature mortality are worrying and highlight health inequalities as this younger group are likely to include a sub-group of vulnerable PWID, who tend to have multi-substance addictions, mental health issues and chaotic lifestyles. Their mortality risk, therefore, is not only from liver causes, but also from accidents, international self-harm and drug-related poisonings. This was evident in our findings, with an almost doubling of risk of death from external causes in the 30–69 age group compared with all adults with HCV. Simmons et al. [24] also found external causes to be the most common cause of death for individuals anti-HCV positive over the same period and drug-related mortality in England has been increasing over the last decade [16]. While DAAs have an important role to play, solutions to address problematic drug use (the most common mode of transmission for HCV in England) are also important to prevent onward HCV spread and to reduce premature mortality from liver and drug-associated deaths.

While clinical studies are required to prove the impact of DAAs on HCV-associated outcomes, they are not sufficient to monitor progress towards the WHO elimination goals. Numerous European countries reported having surveillance of HCV and mortality in a recent WHO survey [27] and as HCV infection is often under-reported of mortality records, linked analysis is required to understand the true burden of HCV-associated disease [24-26]. As Europe works towards the elimination of HCV and progress monitoring is required, this methodology offers a cost-effective option for analysis in countries with suitable datasets.

### Conclusion

Mortality rates, particularly in younger individuals, were higher in those with a diagnosis of HCV infection compared with the general English population, highlighting health inequalities. Our findings provide a baseline of HCV-associated mortality rates pre-DAA and continued monitoring is required as HCV treatments are scaled up to attain HCV elimination ambitions. However, the high mortality rates associated with common co-morbidities, such as problematic drug and alcohol use, highlights the importance of integrated health and social care strategies and commissioning to address the needs of this vulnerable population and reduce inequalities. A holistic approach to drug misuse is required to reduce the double jeopardy of drug and HCV-associated mortality experienced by individuals with HCV infection.
